# Physicochemical and Medicinal Properties of Tualang, Gelam and Kelulut Honeys: A Comprehensive Review

**DOI:** 10.3390/nu13010197

**Published:** 2021-01-10

**Authors:** Datu Agasi Mohd Kamal, Siti Fatimah Ibrahim, Haziq Kamal, Mohd Izhar Ariff Mohd Kashim, Mohd Helmy Mokhtar

**Affiliations:** 1 Department of Physiology, Faculty of Medicine, Universiti Kebangsaan Malaysia, Kuala Lumpur 56000, Malaysia; datuagasi90@gmail.com (D.A.M.K.); timi@ukm.edu.my (S.F.I.); kamalntee1@gmail.com (H.K.); 2 Department of Biomedical Sciences and Therapeutics, Faculty of Medicine and Health Sciences, Universiti Malaysia Sabah, Kota Kinabalu 88400, Malaysia; 3 Centre for Contemporary Fiqh and Sharia Compliance, Faculty of Islamic Studies, Universiti Kebangsaan Malaysia, Bangi 43600, Malaysia; izhar.ukm@gmail.com; 4 Institute of Islam Hadhari, Universiti Kebangsaan Malaysia, Bangi 43600, Malaysia

**Keywords:** Tualang honey, Gelam honey, Kelulut honey, health benefits, anti-oxidative, anti-cancer

## Abstract

Tualang, Gelam and Kelulut honeys are tropical rainforest honeys reported to have various medicinal properties. Studies related to the medicinal properties and physicochemical characteristics of these honeys are growing extensively and receiving increased attention. This review incorporated and analysed the findings on the biological and physicochemical properties of these honeys. Tualang, Gelam and Kelulut honeys were found to possess a wide variety of biological effects attributed to their physicochemical characteristics. Findings revealed that these honeys have anti-diabetic, anti-obesity, anti-cancer, anti-oxidative, anti-microbial, anti-inflammatory and wound-healing properties and effects on the cardiovascular system, nervous system and reproductive system. The physicochemical properties of these honeys were compared and discussed and results showed that they have high-quality contents and excellent antioxidant sources.

## 1. Introduction

Honey has been utilised since ancient times due to its various health benefits. Despite medical advances achieved today, honey remains an integral part of a particular regional demographic. Owing to recent advancements in biotechnology, honey has been revealed to possess newly discovered unique contents and medicinal properties. Stingless bee honey, especially those from Malaysia, Australia, and Brazil, has been recently discovered as a novel source of trehalulose, a biologically active disaccharide with various health benefits [1]. A recent systematic review and meta-analysis showed that honey was superior to usual care for improving upper respiratory tract infections (URTIs). It was also more effective than antibiotics when prescribed to reduce the spread of anti-microbial resistance in URTIs [2]. Honey exhibited superior effects compared with conventional treatments for acute wounds, superficial partial-thickness burns and infected post-operative wounds [3,4]. Some honeys were licensed as a medical product and used in the clinical setting for wound care in Europe and Australia [5].

Tualang honey, Gelam honey and Kelulut honey are tropical rainforest honeys that mostly originated from Malaysia. The health benefits of these honeys have recently been gaining increased attention and popularity, as evidenced by numerous documented studies. Tualang honey is a wild polyfloral honey produced by *Apis dorsata*. This honey is named after one of the tallest tropical rainforest trees, the *Koompassia excelsa* tree (known locally as the Tualang tree), where bees build their hives. The bees collect nectar from plants in the tropical rain forest in the North-eastern region of Peninsular Malaysia in Kedah. This honey possesses anti-microbial, anti-cancer, antioxidant, and anti-inflammatory effects attributed to its high antioxidant contents [6,7]. Gelam honey is also wild honey produced by the *A. dorsata* bees, which build hives on trees locally called Gelam, or *Melaleuca cajuputi Powell*. Gelam honey is mostly obtained from the East Coast of Peninsular Malaysia in Terengganu state. This honey may delay the onset of chronic diseases and infections and regulate the optimal level of enzymatic activity in the body [8]. Kelulut honey is produced by *Trigona* spp., a stingless bee that gathers nectar from polyfloral sources [6,9]. This honey has a slightly sour taste and is more liquid than the other honeys. It possesses antibacterial, antioxidant, and anti-osteoporotic properties due to its high antioxidant properties [10,11].

An updated study to comprehensively analyse the benefits of Tualang, Gelam and Kelulut honey is still required compared to other types of honey such as Manuka honey which has been extensively reviewed. With the recent growth in the body of literature for these kinds of honey, assessment of Tualang, Gelam, and Kelulut honeys is needed to avoid future cross-studies or unnecessary research due to missed reviews of the existing research. In this review, relevant studies related to the medicinal properties, health benefits and physicochemical properties of Tualang, Gelam, and Kelulut honeys were identified and critically analysed. The findings showed that most studies reported the health advantages of honey consumption, whereas some reported disadvantages or no significant changes upon consumption. Furthermore, analyses of physiochemical-related research revealed that Tualang, Gelam and Kelulut honeys have high-quality contents and excellent antioxidant sources.

## 2. Methods of Review

A literature search was undertaken to identify and map out relevant and pertinent articles related to the physicochemical and medicinal properties of Tualang, Gelam, and Kelulut honeys. Peer-reviewed and full-text English articles were gathered from a time frame as early as 1960 to May 2020 in electronic databases, including Scopus, MEDLINE via EBSCOhost, and Google Scholar. A literature search was performed by combining the following set of keywords: (1) Tualang or Kelulut or Gelam and (2) honey. The literature search was further supplemented by referencing related review articles and scientific reports found from the search results.

## 3. Physicochemical Characteristics of Tualang, Gelam, and Kelulut Honeys

Honey from different botanical and geographical origins has different physicochemical properties and compositions that ultimately affect their biological properties [8]. Numerous studies were conducted to investigate the physicochemical properties of Tualang, Gelam and Kelulut honeys. Twenty-two studies related to the physicochemical and content analyses on these selected honeys were found. These honeys have unique physicochemical properties mostly attributed to Malaysia’s tropical climate. Table 1; Table 2 show the physicochemical characterisation of Tualang, Gelam and Kelulut honeys extracted from various studies. Considering Kelulut honey is a polyfloral honey, this review only included studies on Kelulut honey that mainly originated from the *Acacia mangium* trees to obtain a fair comparison.

On the one hand, Tualang and Gelam honeys mostly complied with the accepted range by the two most common legislation of honey criteria and standards referred to as the European Honey Legislation [12] and Codex Alimentarius Standards for Honey [13]. On the other hand, the Kelulut honey in various studies followed the Malaysian Standard Kelulut (Stingless Bee) [14]. According to the European Honey Legislation and the Codex Alimentarius Standards, honey moisture should be less than 20%, with glucose and fructose composition of more than 60 g/100 g, sucrose content of not more than 5 g/100 g and electrical conductivity of not more than 0.8 mS/cm. According to the Malaysian Standard Kelulut, the raw honey moisture content from stingless bee must be less than 35 g/100 g, with a pH of less than 3.8 and 5-hydroxymethylfurfural of less than 30 mg/kg.

Most of the studies reported that Tualang and Gelam honeys contained more than 20% moisture content, thus violating European Honey Legislation and Codex Alimentarius Standards. Nonetheless, honey samples from tropical countries, such as Malaysia, typically have higher moisture content, which could be due to the rainy season all over the year [15]. Therefore, Malaysia’s honey is always first treated by evaporation to reduce the water content, thereby simultaneously increasing the honey quality [16].

Meanwhile, the electrical conductivity of honey is used to determine the botanical origin and purity of honey [17]. An electrical conductivity of less than 0.8 mS/cm indicates blossom honey, whilst more than 0.8 mS/cm indicates honeydew honey [18]. According to the European Honey Legislation and the Codex Alimentarius Standards, honey’s electrical conductivity should not be more than 0.8 mS/cm. However, the Malaysian Standard Kelulut has not determined any range for an acceptable limit of electrical conductivity. The electrical conductivity of Tualang, Gelam and Kelulut honeys reported in this review is in a broad range of 0.74–1.51 mS/cm. Various factors, such as storage, time, temperature, water content and concentration of ions and minerals, were reported to contribute to the electrical conductivity of honey [16].

Most bacteria grow in a neutral and mildly alkaline environment, whereas yeasts and moulds could grow in an acidic environment (pH = 4.0–4.5). Conversely, the pH values of honey are neither those needed for bacteria nor yeast growth [15]. This is of great importance during storage, as they influence the texture, stability and shelf-life of honey [18]. The pH value of Tualang, Gelam, and Kelulut honeys were reported to be in the range of 3.14–3.83. Moreover, the sugar content of these three honeys has complied with the requirement of the European Honey Legislation and Codex Alimentarius Standards. In terms of honey colour characteristics, Tualang honey is described as light amber honey and Gelam honey belongs to dark amber honey, whilst standard Kelulut honey is amber brown [15,19].

Many studies found that the antioxidant capacity of honey is strongly correlated with the concentration of its phenolic contents [20,21]. The phenolic content of honey comes from the plant that the bees feed on [22]. Thus, botanical origin plays a significant role in determining the constituents and antioxidant activity of honey [23]. Colour intensity is also associated with antioxidant properties. Numerous studies, including those on Gelam, Tualang and Kelulut honey, associated the honey colour intensity with antioxidant properties. Darker-coloured honey showed higher antioxidant activity and higher total phenolic content [22,24,25,26]. The present review also found that these three honeys have a high content of phenolic and flavonoid. Amongst the reviewed studies comparing the antioxidant properties of Tualang, Gelam and Kelulut honey, one study showed that Kelulut honey has the highest antioxidant properties in ranking after Manuka honey amongst Gelam and Tualang honeys [25]. By contrast, another study found Tualang honey to have higher antioxidant properties than Manuka, Kelulut, and Gelam honeys [27]. As mentioned earlier, the study also revealed higher free radical scavenging and antioxidant activity in Tualang honey than in Gelam and Borneo honeys [26].

The determination of trace elements in honey is essential for quality control and monitoring of trace element composition. High levels of trace elements could be dangerous and cause toxicity. The levels of trace elements in Tualang, Gelam and Kelulut honeys were within the permissible value set by the World Health Organization (WHO), and no honey contamination with pesticide residues was found [28].

**Table 1:** Physicochemical characteristics of Tualang, Gelam and Kelulut honeys.

Physicochemical Characteristics	Tualang Honey	Gelam Honey	Kelulut Honey
Colour characteristic (mm Pfund)	74.00–113.0 [15,16,29]	122.00–139.0 [15,16,30]	1.25 [31]
pH	3.14–3.80 [15,16,29,32]	3.38–3.83 [15,16,30,32]	3.27–3.30 [31,33]
Moisture content	17.53–26.51% [15,16,29,32]	17.93–26.50% [15,16,30,32]	21.52–31% [31,33,34]
Electrical conductivity, (mS/cm)	0.75–1.37 [15,16,29]	0.74–1.51 [15,16,30]	0.33–0.69 [35]

**Table 2:** Chemical constituents of Tualang, Gelam and Kelulut honeys.

Chemical Constituent	Tualang Honey	Gelam Honey	Kelulut Honey
Total sugar content (g/100 g)	63.60–72.94 [29,32,36]	64.93–72.57 [30,32,36]	65.83–71.65 [31,33,34,36]
Reducing sugar (g/100 g)	61.94 [29]	62.17 ± 0.73 [30]	No report found
Sucrose (g/100 g)	˂0.01–1.66 [29,32,36]	˂0.01–2.77 [30,32]	˂0.01–32.30 [31,33,35,36]
Glucose (g/100 g)	30.07–47.134 [32,36]	32.85–50.447 [32,36]	9.22–23.44 [31,33,35,36]
Fructose (g/100 g)	41.732–44.56 [32,36]	44.908–44.74 [32,36]	15–22.05 [31,33,35,36]
Maltose (g/100 g)	4.491 [32]	1.291 [32]	0–27.41 [31,33]
Protein content (g/kg)	3.6–6.6 [29,32,36]	3.14–7.0 [30,32,36]	3.9–8.5 [33,34,36]
Proline content (mg/kg)	248.53 ± 1.33 [29]	261.33 ± 1.33 [30]	No report found
Mineral content (mg/kg)			
Sodium	268.23–704.5 [28,36]	196.84–666.0 [28,36]	589.7 [36]
Potassium	976.9–1576.40 [28,36]	1132.2–1363.40 [28,36]	732.2 [36]
Calcium	76.4–165.10 [28,36]	177.9–275.77 [28,36]	191.9 [36]
Iron	11.17–128.13 [28,36]	8.45–142.37 [28,36]	6.57 [36]
Magnesium	35.03–71.04 [28,36]	31.63–49.38 [28,36]	33.81 [36]
Zinc	2.28–13.20 [28,36]	1.45–29.23 [28,36]	2.162 [36]
Arsenic	0.051–0.062 [28,36]	0.064–0.070 [28,36]	0.019 [36]
Lead	0.183–0.231 [28,36]	0.145–0.777 [28,36]	0.154 [36]
Cadmium	0–0.004 [28,36]	0.005–0.05 [28,36]	0.002 [36]
Copper	1.25–2.144 [28,36]	2.089–2.21 [28,36]	1.776 [36]
Cobalt	0.033 ± 0.002 [28]	0.082 ± 0.005 [28]	No report found
Total phenolic content mg/kg (gallic acid)	251.7–1103.94 [16,21,29,37,38]	606.17–1597.43 [16,21,38]	477.30–614.7 [31,33]
Total flavonoid content	49.04–185.11 (rutin) [16,21] 65.65 (catechin) [29] 504.5 (quercetin) [38]	183.43–328.86 (rutin) [16,21] 461.1 (quercetin) [38]	36.3 (quercetin)

## 4. Medicinal Properties of Tualang, Gelam and Kelulut Honeys

**Figure 1:**
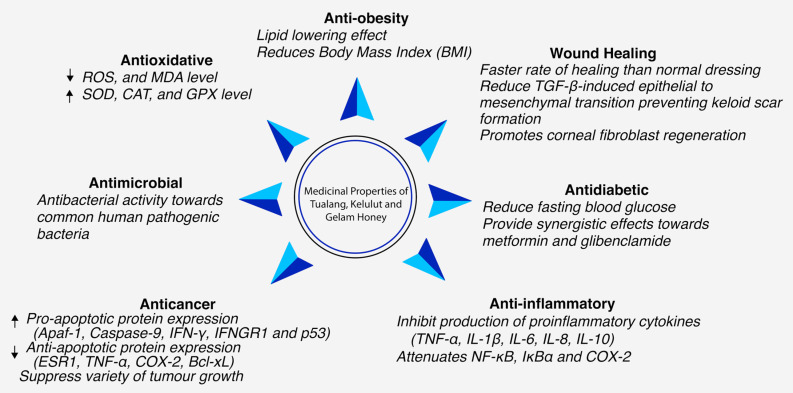
The medicinal properties of Tualang, Gelam and Kelulut honeys. ROS: reactive oxygen species; MDA: malondialdehyde; SOD: superoxide dismutase; CAT: catalase; GPX: glutathione peroxidase; TGFβ: transforming growth factor beta; Apaf-1: Apoptotic protease activating factor-1; IFN-γ: interferon-gamma; IFNGR1: interferon gamma receptor-1; p53: tumor protein P53; ESR1: oestrogen receptor-1; TNF-α: tumor necrosis factor alpha; Bcl-xL: B-cell lymphoma-extra-large; IL-1β: interleukin-1 beta; IL-6: interleukin 6; IL-8: interleukin 8; IL-10: interleukin 10; NF-kB: nuclear factor kappa B; IκBα: NF-kappa-B inhibitor alpha; COX-2: cyclooxygenase-2.

In this section, the medicinal properties of Tualang, Gelam and Kelulut honeys are discussed in accordance with different properties. A total of 99 articles regarding the medicinal properties of Tualang, Gelam and Kelulut honeys were obtained, including 59 in vivo studies, 25 in vitro studies, 2 ex vivo, one study combine in-viva and invitro technique and 12 clinical trials involving humans. Among medicinal properties discussed below, all three honey shared these major health benefits; (a) anti-oxidative; (b) anti-cancer; (c) anti-inflammatory; (d) wound-healing; (e) anti-microbial; (f) anti-diabetic and; (g) anti-obesity (as seen in Figure 1).

### 4.1. Anti-Oxidative Properties

**Figure 2:**
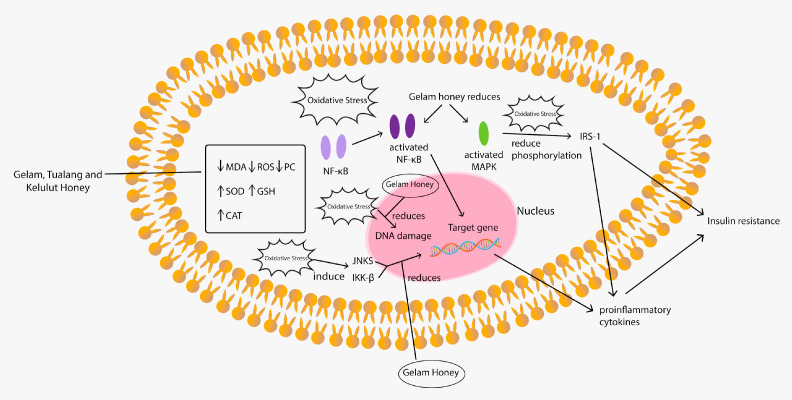
Anti-oxidative mechanism of Tualang, Gelam and Kelulut honeys. ROS: reactive oxygen species; MDA: malondialdehyde; SOD: superoxide dismutase; CAT: catalase; GSH: glutathione; PC: protein carbonyl; NF-kB: nuclear factor kappa B; MAPK: mitogen-activated protein kinases; JNKS: c-jun N-terminal kinases; IKK-β: I-kappa-B-kinase beta; IRS-1: insulin receptor substrate-1.

One of the most well-known benefits of honey is its anti-oxidative property. In this review, the most positive medicinal effects of Malaysian honey were attributed to its antioxidant properties. A plethora of studies regarding the antioxidative characteristic and pathways of Tualang, Gelam and Kelulut honeys are summarised and illustrated in Table 3 and Figure 2, respectively. Two studies reported that Gelam honey reduced oxidative damage by reducing DNA damage and the malondialdehyde (MDA) level of young, middle-aged and aged rats by modulating antioxidant enzyme activities [39,40]. Another study showed that the combination of Gelam honey and ginger provided a better antioxidant effect than Gelam honey or ginger alone, as evidenced by the significantly reduced superoxide dismutase (SOD) and catalase (CAT) activities, reduced MDA level, increased (reduced glutathione) GSH level and increased GSH/glutathione disulfide (GSSG) ratio in diabetic rats [41]. Another separate study reported the protective effect of Gelam honey on human diploid fibroblast (HDFs) exposed to gamma rays. Gamma irradiation to HDFs decreased SOD, CAT, and glutathione peroxidase (GPx) gene expression levels and enzyme activities, whilst treatment with Gelam honey increased both parameters [42].

In diabetes, oxidative stress interrupts glucose metabolism’s normal coupling to insulin secretion by activating stress signaling nuclear factor-κB (NF-κB) and p38 mitogen-activated protein kinase (MAPK) pathways. The activation of NF-κB causes the production of inflammatory cytokines that promote insulin resistance whilst activating MAPK to reduce insulin receptor substrate-1 (IRS-1) serine phosphorylation, thereby causing insulin resistance [43,44]. A series of studies have proven Gelam honey’s protective effect on diabetes through this insulin resistance signaling pathway mechanism. Gelam honey exerts protective effects against diabetes and hyperglycemia-induced oxidative stress through improving insulin content and insulin resistance by producing the differential expression of insulin signaling pathways MAPK, NF-κB, IRS-1 (ser307) and Akt in HIT-T15 cells [45]. Gelam honey had been shown to attenuate the oxidative stress-induced inflammatory pathways when pretreatment with Gelam honey or quercetin reduced the expression of phosphorylated jun-N-terminal kinase, IkappaB kinase and IRS-1, thereby significantly reducing the expression of proinflammatory cytokines, such as tumour necrosis factor alpha (TNF-α), interleukin-6 (IL-6) and interleukin-1β (IL-1β). A significant increase in phosphorylated Akt was also found, indicating the protective effects against inflammation and insulin resistance [46]. Batumalaie et al. (2014) reported that pretreatment of HIT-T15 cells with Gelam honey before culturing in hyperglycemic media yielded a significant decrease in the production of reactive oxygen species (ROS), glucose-induced lipid peroxidation and a significant increase in insulin content and the viability of cells cultured under hyperglycemic condition [47]. This series of studies demonstrated the potential anti-oxidative role of Gelam honey in managing diabetes through improving pathway-related insulin resistance and production.

Other studies have also explored the antioxidant capacity of Tualang honey. In animal study, Tualang honey was reported as having a hypoglycemic effect with reduced elevated MDA levels. It restored SOD and CAT activities in the pancreas of diabetic rats [48], suggesting the hypoglycemic effect of Tualang honey through its anti-oxidative effect on the pancreas. By contrast, another study using glibenclamide- and metformin-treated diabetic rats yielded no significant thiobarbituric acid reactive substances (TBARS) and CAT effects except that in GPx. However, the authors reported that a combination of glibenclamide, metformin and Tualang honey significantly up-regulated the CAT activity, down-regulated the GPx activity and reduced TBARS [49]. These findings suggested that Tualang honey potentiates the effect of glibenclamide and metformin to protect diabetic rat pancreas against oxidative stress and damage. Another study on diabetic rats revealed that Tualang honey ameliorated oxidative stress in kidneys of diabetic rats [50] and potentiated the effect of glibenclamide and metformin to protect diabetic rat kidneys against oxidative stress [51]. An in vitro study showed that Tualang honey improved human corneal epithelial progenitor cell migration and cellular resistance to H_2_O_2_-induced oxidative stress [52]. However, a study investigating the anti-inflammatory and antioxidant effects of Tualang honey in alkali injury on the eyes of rabbits did not find any significant difference between the Tualang honey-treated group and the group who underwent conventional treatment in terms of inflammatory feature and antioxidant status [53]. Consistent with the previous findings, Ahmad et al. (2017) reported the anti-oxidative properties of Tualang honey in a human study. The authors stated that consumption of 1.5 (high dose) and 0.75 (low dose) g/kg of Tualang honey demonstrated a comparable response in increasing antioxidant activity (ferric reducing antioxidant power) and suppressing oxidative stress (MDA and ROS) in female athletes. The time-course effect of Tualang honey that provides optimal antioxidant activity and oxidative stress protection was between 1 and 2 hours after its consumption [54].

Kelulut honey has also been demonstrated to have anti-oxidative properties when it ameliorated glucocorticoid-induced osteoporosis via a reduction in lipid peroxidation and increase in SOD [11]. Kelulut honey supplementation prevented sperm and testicular oxidative damage in streptozotocin-induced diabetic rats by increasing SOD activity and GSH level and decreasing protein carbonyl (PC) and MDA levels in the sperm and testis [55].

The antioxidant properties of Gelam honey were well demonstrated by animal and in vitro studies discussed above. However, future works should explore the anti-oxidative effect of Gelam honey on humans. The antioxidant properties of honey have been attributed to its phytochemical content, which is phenolic and flavanoid. A study reported that the antioxidant activity of several types of honey originating from different countries depends on the concentration of phenolic and flavonoid groups [56]. These phenolic and flavonoids were reported to derive from the plant that the bees feed on [57]. The proposed honey antioxidant action mechanisms include free radical sequestration, hydrogen donation, metallic ion chelation, flavonoid substrate action for hydroxyl and superoxide radical actions [58].

**Table 3:** Summary of anti-oxidative properties.

Type of Honey	Type of Study	Findings	References
Tualang honey	In vivo	Tualang honey reduced elevated malondialdehyde (MDA) levels and restored superoxide dismutase (SOD) and catalase (CAT) activities in diabetic rats.	[48]
Tualang honey	In vivo	Tualang honey potentiated the effect of glibenclamide and metformin to protect diabetic rat pancreas against oxidative stress and damage.	[49]
Tualang honey	In vivo	Combination of Tualang honey with metformin or glibenclamide provided additional antioxidant effect to these drugs toward diabetic rat kidneys.	[51]
Kelulut honey	In vivo	Kelulut honey reduced oxidative stress by reducing lipid peroxidation and increasing SOD. It also maintained bone structure, increased osteoblast and reduced osteoclast number in glucocorticoid-induced osteoporosis rat.	[11]
Gelam honey	In vivo	Gelam honey reduced oxidative damage in young and middle-aged rats by modulating antioxidant enzyme activities.	[39]
Gelam honey	In vivo	Gelam honey reduced oxidative damage in young and aged rats through modulation of antioxidant enzyme activities.	[40]
Gelam honey	In vivo	Combination of Gelam honey and ginger provided a better antioxidant effect than Gelam honey or ginger alone, as evidenced by significantly reduced SOD and CAT activities, depleted MDA level, increased glutathione (GSH) level and increased GSH/glutathione disulfide (GSSG) ratio in diabetic rats.	[41]
Gelam honey	In vitro	Gelam honey and quercetin attenuated the oxidative stress-induced inflammatory and insulin signalling pathways in pancreatic hamster cells.	[46]
Gelam honey	In vitro	Gelam honey-induced differential expression of mitogen-activated protein kinases (MAPK), nuclear factor kappa B (NF-κB), insulin receptor substrate-1 (IRS-1) (ser307), and Akt in HIT-T15 cells showed that Gelam honey exerted protective effects against diabetes and hyperglycaemia-induced oxidative stress by improving insulin content and insulin resistance.	[45]
Tualang honey	In vitro	Tualang honey possessed antioxidant properties and could improve cell migration and cellular resistance to oxidative stress in human corneal epithelial progenitor cells in vitro.	[52]
Gelam honey	In vitro	Gelam honey modulated the expression of antioxidant enzymes at gene and protein levels in irradiated human diploid fibroblasts, suggesting its potential as a radioprotectant agent.	[42]
Gelam honey	In vitro	Pre-treatment of cells with Gelam honey extract or its flavonoid components before culturing in hyperglycaemic condition showed a significant decrease in reactive oxygen species (ROS) production and glucose-induced lipid peroxidation and a significant increase in insulin content and the viability of pancreatic cells.	[47]
Tualang honey	Human study	Consumption of high (1.5 g/kg) and low (0.75 g/kg) doses of Tualang honey demonstrated a comparable response in increasing antioxidant activity and suppressing oxidative stress in female athletes. The time-course effect of Tualang honey that provided optimal antioxidant activity and oxidative stress protection was between 1 and 2 hours after its consumption.	[54]
Tualang honey	In vivo	No significant difference between Tualang honey treatment and conventional treatment in terms of inflammatory feature and antioxidant status in alkali injury on the eyes of rabbits.	[53]

### 4.2. Anti-Cancer Properties

**Figure 3:**
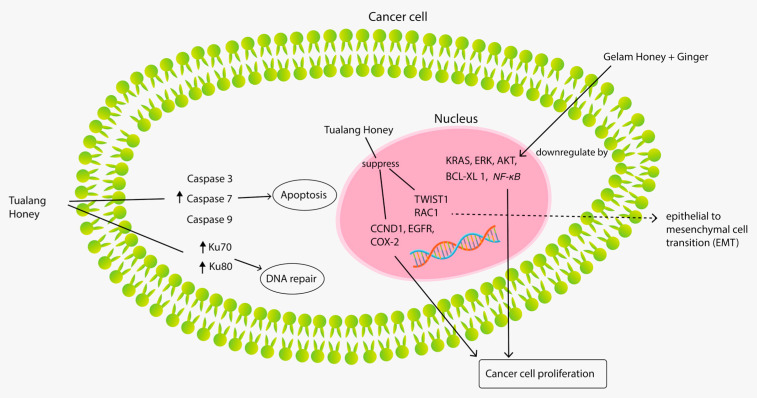
Anti-cancer mechanism of Tualang, Gelam and Kelulut honeys. KRAS: K-Ras encoding gene; ERK: extracellular signal-regulated kinase; AKT: protein kinase B; Bcl-xL: B-cell lymphoma-extra-large; NF-kB: nuclear factor kappa B; TWIST1: Twist-related protein 1 gene; RAC1: Ras-related C3 botulinum toxin substrate 1 gene; CCND1: Cyclin D1 gene; EGFR: epidermal growth factor receptor gene; COX-2: cyclooxygenase-2.

Most of the literature on Tualang, Gelam, and Kelulut honeys focused on their anti-cancer properties in many models and various mechanisms. Studies involved all sets of subjects, from animal to cell culture and human studies. A total of 18 studies were found: five animal studies, 11 in vitro studies and two human studies. A summary of the anti-cancer properties for Tualang, Gelam, and Kelulut honey is provided in Table 4, while their anti-cancer pathway is illustrated in Figure 3.

Three in-vivo studies demonstrated the anti-cancer properties of Tualang honey in a breast cancer model [59,60,61]. The anti-cancer effect of Tualang honey was demonstrated through modulation in five aspects: tumor growth, tumor grading and haematologic, oestrogenic and apoptotic activities. Lower growth rate, tumor size, weight and multiplicity of tumor in the Tualang honey-treated group than those in the untreated control were reported in a rat breast cancer model [59,60,61]. Histologically, breast cancer rats treated with Tualang honey were primarily graded I and II compared with the untreated control, in which the majority was grade III [59,60]. Furthermore, Tualang honey increased the expression of proapoptotic proteins (Apaf-1, caspase-9, IFN-γ, IFNGR1, and p53) and decreased the expression of antiapoptotic proteins (ESR1, TNF- α, COX-2 and Bcl-xL) [59,61]. Tualang honey treatment was also found to modulate haematological parameters, such as haemoglobin (Hb), red blood cells (RBCs), packed cell volume (PCV), mean corpuscular volume (MCV), red cell distribution width (RDW), mean corpuscular hemoglobin concentration MCHC, polymorphs and lymphocytes values [59]. Manuka honey was reported to have the same anti-cancer properties as Tualang honey in an animal breast cancer model [61].

The anti-cancer effect of Tualang honey was successfully demonstrated in another three in vitro studies using the human breast cancer cell line [62,63,64]. These studies have shown that Tualang honey treatment-induced caspase-3, caspase-7 and caspase-9 activation and decreased the mitochondrial membrane potential in all tested cancer cells, indicating that Tualang honey-induced apoptosis occurred least via a mitochondrial-dependent pathway [62,63]. Depolarisation of the mitochondrial membrane in breast cancer cell lines increased when Tualang honey was used in conjunction with tamoxifen compared with treatment with tamoxifen alone [63]. Interestingly, Tualang honey was found to be cytotoxic to breast cancer cell line (MCF-7); it also protected non-tumorigenic epithelial breast cell line (MCF-10A) from the toxic effects of tamoxifen active metabolite 4-hydroxytamoxifen by increasing the efficiency of the DNA repair mechanism in these cells, as evidenced by the increment in DNA repair proteins Ku70 and Ku80 [64]. Therefore, clinical trials may focus on Tualang honey as a possible adjuvant to be used with tamoxifen to minimise tamoxifen dosage and thus minimise the adverse effects. In a clinical trial, the combination of Tualang honey and anastrozole in decreasing breast background parenchymal enhancement in breast cancer patients was more successful than anastrozole alone [65]. Three-tier evidence from animal, in vitro and human studies demonstrated the benefit of Tualang honey in breast cancer, highlighting the potential use of Tualang honey as a natural supplement to chemotherapeutic agents used for breast cancer treatment.

In an animal model of oral squamous cell carcinoma, Tualang honey showed chemopreventive capabilities by suppressing cancer cell proliferation and activity, as demonstrated by the decrease in the expression of CCND1, EGFR, and COX-2, which are genes related to cancer development and cellular proliferation. In addition, Tualang honey inhibited the aggressiveness of oral squamous carcinoma by down-regulating TWIST1 and RAC1, which are the genes representing epithelial-to-mesenchymal transition (EMT), and overexpressing β-catenin and E-cadherin [66]. Meanwhile, another study proved that Tualang honey has an anti-proliferative effect on oral squamous cell carcinoma and osteosarcoma cell lines by inducing early apoptosis [67].

Other various anti-cancer potentials of Tualang honey have been discovered through in vitro studies, including anti-cancer activity against cervical cancer cell lines; this activity shares the same mechanism as in the in vitro study on breast cancer cell lines discussed above [62]. Man et al. (2018) reported the antileukemic effect of Tualang honey through its apoptosis-inducing ability for acute and chronic myeloid leukemia cell lines [68]. In another in vitro study, Tualang honey protected keratinocytes from ultraviolet radiation-induced inflammation and DNA damage by modulating the early biomarkers of photocarcinogenesis, thus providing significant protection from the adverse effects of ultraviolet B (UVB) radiation and a suggestion for its photochemopreventive potential [69]. A randomised clinical trial using Tualang honey has been conducted on cancer-related fatigue in patients with head and neck cancer. These patients received either 20 mg of Tualang honey or 100 mg of vitamin C tablet for 8 weeks, in which the level of fatigue and quality of life were measured using questionnaires with additional measurement of white cell count and C-reactive protein level. No significant changes were detected between the vitamin C and Tualang honey groups for the white cell count and C-reactive protein. However, the Tualang honey group showed a substantial improvement in fatigue and quality of life compared with the vitamin C group [70]. The author suggested that Tualang honey could be used as a supplement to improve the quality of life and reduce fatigue in patients with head and neck cancer.

Anti-cancer properties were also exhibited by Kelulut and Gelam honeys. A study by Yazan et al. (2017) showed that 1183 mg/kg of Kelulut honey treatment twice daily for 8 weeks significantly reduced the total number of aberrant crypt foci, aberrant crypts and crypt multiplicity in colorectal cancer-induced rats [71]. Several studies have demonstrated the anti-proliferative and apoptotic induction properties of Gelam honey on liver, colorectal and colon cancer cell lines [72,73,74]. A combination of Gelam honey and ginger produced synergism that provided an anti-cancer activity toward colon cancer cell through stimulation of early apoptosis (upregulation of caspase-9 and IκB genes) accompanied by downregulation of the expression of KRAS, extracellular signal-regulated kinase (ERK), protein kinase B (Akt), B-cell lymphoma-extra-large (Bcl-xL), and nuclear factor kappa B (NFkB) genes, which are related to cancer cell proliferation [75]. This combination also provided anti-cancer effects toward colorectal cancer by inhibiting mammalian target of rapamycin (mTOR) and Wnt/β catenin signaling pathways and the induction of apoptosis [76].

The combination of Gelam honey and ginger potentiated the anti-cancer effect of 5-fluorouracil against HCT 116 colorectal cancer cells by reducing proliferation and increasing apoptosis [73]. In a separate study, Gelam honey combined with 5-fluorouracil also provided a synergistic cytotoxic effect against human adenocarcinoma colon cancer HT-29 cells. The combined treatment showed a significantly higher cytotoxic effect and apoptosis induction on HT-29 cells than the stand-alone treatment with Gelam honey and 5-fluorouracil [74]. Another study showed that gamma irradiation up-regulated ATM, p53, p16^ink4a^ and cyclin D1 genes and subsequently initiated cell cycle arrest at the G0/G1 phase and induced apoptosis. However, pre-treatment with Gelam honey caused downregulation of these genes in irradiated HDFs. Gelam honey treatment caused cells to progress to the S phase with fewer cells in the G0/G1 phase, whilst apoptosis was inhibited [77]. This finding suggested the potential of Gelam honey as a radioprotector agent against gamma irradiation. Another study showed that Gelam honey inhibited the proliferation of HT 29 colon cancer cells by inducing DNA damage and apoptosis and suppressing inflammation [78]. In vitro studies have successfully revealed the anti-cancer properties of Gelam honey or in combination with ginger and their potentiating effect toward 5-fluorouracil. Thus, human studies using Gelam honey as a supplement to 5-fluorouracil should be conducted to prove the anti-cancer benefit of Gelam honey toward liver, colorectal and colon cancer cell lines as discussed above.

Honey generally contains various kinds of phytochemical with high phenolic and flavonoid content, thus contributing to its high antioxidant activity [79]. An agent with potent antioxidant properties could potentially prevent cancer, as free radicals induce oxidative stress, which causes cancer formation [80]. Moreover, a different type of polyphenol found in honey was reported to have anti-cancer properties against several types of cancer through various mechanisms. These polyphenols include caffeic acid, caffeic acid phenyl esters, chrysin, galangin, quercetin, kaempferol, acacetin, pinocembrin, pinobanksin and apigenin [81]. Honey is a natural immune booster, a natural anti-inflammatory agent, a natural anti-microbial agent, a natural promoter for chronic ulcers and wound healing and a natural antioxidant; these all serve as a basis for its anti-cancer properties.

**Table 4:** Summary of anti-cancer properties.

Type of Honey	Type of Study	Findings	References
Kelulut honey	In vivo	Kelulut honey possessed chemo-preventive properties in rats induced with colorectal cancer and it was found to be not toxic to rats.	[71]
Tualang honey	In vivo	Tualang honey ameliorated breast cancer by increasing the susceptibility of proapoptotic proteins; apoptotic protease activating factor-1 (Apaf-1) interferon-gamma (IFN-γ) interferon gamma receptor-1 (IFNGR1) tumor protein P53 (p53) and decreased the expression of anti-apoptotic proteins; tumour necrosis factor alpha (TNF-*α*), cyclooxygenase-2 (COX-2) and B-cell lymphoma-extra-large (Bcl-xL).	[61]
Tualang honey	In vivo	Tualang Honey alleviated breast cancers in rats by reducing cancer cell growth and enhanced histological grading.	[60]
Tualang honey	In vivo	Tualang honey showed chemo-preventive properties in oral squamous cell carcinoma-induced rats by suppressing cancer cell proliferation and activity and preserving cellular adhesion.	[66]
Tualang honey	In vivo	Tualang Honey alleviated breast carcinogenesis by modulating haematologic, oestrogenic and apoptotic activities in the breast cancer animal model.	[59]
Tualang honey	In vitro	Tualang honey promoted apoptotic cell death induced by tamoxifen in breast cancer cell lines.	[63]
Gelam honey	In vitro	Gelam honey exhibited anti-proliferative activity and apoptotic induction in liver cancer cell line.	[72]
Tualang honey	In vitro	Tualang honey showed an anti-proliferative effect on oral squamous cell carcinoma and osteosarcoma cell lines by inducing early apoptosis.	[67]
Gelam honey	In vitro	Gelam honey acted as a radioprotector against gamma irradiation by attenuating radiation-induced cell death in human diploid fibroblasts.	[77]
Gelam honey combine with ginger	In vitro	Combined treatment of Gelam honey and ginger extract potentiated the anti-cancer effect of fluorouracil against colorectal cancer cells.	[73]
Gelam and Nenas honey	In vitro	Gelam and Nenas honeys are capable of suppressing HT 29 colon cancer cell growth by inducing apoptosis and suppressing inflammation.	[78]
Tualang honey	In vitro	Tualang honey demonstrated cytotoxic and apoptotic activities against human breast and cervical cancer cell lines with the mitochondrial apoptotic pathway’s involvement.	[62]
Gelam honey	In vitro	Gelam honey showed a synergistic cytotoxic effect with 5-fluorouracil against human adenocarcinoma colon cancer HT-29 cells.	[74]
Gelam honey combined with ginger	In vitro	Combined treatment of ginger and Gelam honey was more effective than treatments with either Gelam honey only or ginger only in inhibiting the growth of HT29 colon cancer cells by inducing early apoptosis, modulating the expression of genes involved in the KRAS/ERK/ PI3K/AKT pathways and suppressing inflammation via the NFκB pathway.	[75]
Tualang honey	In vitro	Tualang honey demonstrated apoptosis-inducing ability for acute and chronic myeloid leukaemia (K562 and MV4-11) cell lines.	[68]
Tualang honey	In vitro	Tualang honey protected keratinocytes from ultraviolet radiation-induced inflammation and DNA damage via modulation in early biomarkers of photocarcinogenesis.	[69]
Tualang honey	In vitro	Tualang honey was found to be cytotoxic to breast cancer cell line (MCF-7) but protected non-tumorigenic epithelial breast cell line (MCF-10A) from the toxic effects of tamoxifen active metabolite 4-hydroxytamoxifen.	[64]
Tualang honey	Human study	Tualang honey improved cancer-related fatigue and quality of life of patients with head and neck cancer post-chemotherapy or radiotherapy.	[70]
Tualang honey	Human study	Combination of Tualang honey and anastrozole showed more improvement in decreasing breast background parenchymal enhancement in patients with breast cancer than anastrozole alone.	[65]

### 4.3. Anti-Microbial Properties

The antibiotic resistance of bacteria is increasing nowadays; thus, the discovery of alternative antibacterial agents is urgently needed. Honey has been reported in numerous studies to possess anti-microbial activity. Although the anti-microbial activity of honey has been effectively established against an extensive spectrum of microorganisms, it differs depending on the type of honey. The anti-microbial activity of Tualang, Gelam, and Kelulut honeys has been documented in various studies. Table 5 provides a summarisation of the anti-microbial properties of these honeys. Tualang honey has been reported to possess antibacterial activity against *Salmonella Typhi*, *Streptococcus pyogenes*, *Staphylococcus aureus*, *Staphylococcus epidermidis*, *Enterococcus faecium*, *Enterococcus faecalis*, *Escherichia coli*, *Salmonella enterica serovar Typhimurium*, *Klebsiella pneumoniae*, and vancomycin-resistant enterococci [82,83].

The antibacterial mechanism of honey is attributed to its high osmolarity, acidity and content of H_2_O_2_ and non-peroxide components [84]. H_2_O_2_ is derived from the hydrolysation of glucose-by-glucose oxidase, which could only occur when honey is diluted. H_2_O_2_ level is determined by relative glucose oxidase levels synthesised by the bee and catalase derived from floral [85]. In pure honey, high osmotic pressures and high acidity are responsible for the antibacterial properties of honey at this stage [84]. Some types of honey, such as Manuka honey, possess high non-peroxide antibacterial activity that could retain the antibacterial activity even after treatment with catalase [86]. They are known as active non-peroxide honey, containing various non-peroxide components that possess antibacterial actions [9]. These components include phenolic acids, flavonoids, methylglyoxal, and methyl syringate [84]. The antibacterial potencies of Tualang, Gelam, and Kelulut honeys were generally comparable to those of Manuka honey, with Tualang honey being the closest. Gelam, Kelulut, and Tualang honeys have a high antibacterial activity of total and non-peroxide activities, indicating that peroxide and non-peroxide components are mutually important antibacterial mechanism of these honeys [9]. Another study looked into the relationship between the phytochemicals and the antibacterial activity in wild honey of Sabah, Malaysia. Yap et al. (2012) found that anti-microbial activity was strongly positively correlated with the phenolic content, whilst the total flavonoid content was moderately positively correlated with antibacterial activity [87].

Gelam and Tualang honeys have been found to contain *Lactobacillus acidophilus* with anti-microbial activity against multiple antibiotic-resistant *S. aureus*, *Staphylococcus epidermis* and *Bacillus subtilis*. The anti-microbial compounds found were stable to heating, proteolytic enzyme treatment and pH adjustments [88]. This finding further strengthened the presence of anti-microbial properties in Tualang and Gelam honeys. A study comparing the anti-bacterial effects of topical gentamicin, topical Tualang honey and a combination of the two on pseudomonas-induced keratitis in rabbit eyes showed similar clinical and anti-microbial effects in all groups [89]. However, these results could not be verified because the study utilised a low sample size and no control group.

Two comparative studies on antibacterial potency revealed comparable results against Gram-negative bacteria tested with Aquacel-Manuka honey and Aquacel-Tualang honey dressing on the burn wound. However, for Gram-positive bacteria, Tualang honey was not as effective as the usual care products such as silver-based dressing or medical grade and Aquacel-Manuka honey dressings [90]. In another comparison study, Tualang honey showed equivalent or better activities than Manuka honey in some wound and enteric microorganisms, especially against *Stenotrophomonas maltophilia* and *Acinetobacter baumannii* [91]. Meanwhile, a study comparing the antibacterial potency amongst Gelam, Tualang and Durians honey showed that Gelam honey possessed the highest antibacterial effect [83].

Apart from its effective antibacterial property, honey possesses antifungal and antiviral properties. Tualang honey administration, especially at 40 and 60 g daily doses for 6 months in patients with asymptomatic human immunodeficiency virus (HIV), was shown to reduce viral load and improve their CD4 counts and quality of life [92]. *Pediococcus acidilactici*, a lactic acid bacteria strain, was isolated from Tualang honey and possessed antifungal activity against pathogenic Candida species [93].

The emergence of antibiotic-resistant bacteria has become a major problem among healthcare practitioners. Honey seems to be a promising alternative to solve this problem. As discussed in this section, Tualang and Gelam honeys were reported to be effective against a wide range of multi-resistant bacteria, indicating the possible utilisation of honey to combat antibiotic-resistant bacteria. Even though the current findings presented a strong case for honey’s anti-microbial properties, future works should explore anti-microbial resistance toward honey and its side effects to be safely utilised as an alternative or to provide a synergistic effect with anti-microbial therapy [9,94].

**Table 5:** Summary of anti-microbial properties.

Type of Honey	Type of Study	Findings	References
Tualang honey	In vivo	Topical gentamicin, topical Tualang honey and the combination of the two all showed similar clinical and anti-microbial effects in treating Pseudomonas-induced keratitis in rabbits.	[89]
Tualang and Gelam honey	In vitro	Tualang and Gelam honeys showed antibacterial activity against common human pathogenic bacteria, namely *Staphylococcus aureus*, *Staphylococcus epidermidis*, *Enterococcus faecium*, *Enterococcus faecalis*, *Escherichia coli*, *Salmonella enterica serovar Typhimurium* and *Klebsiella pneumoniae*, including vancomycin-resistant enterococci, with Gelam honey showing the highest antibacterial effect amongst the tested Malaysian honey samples.	[83]
Tualang honey	Human	Tualang honey demonstrated bactericidal and bacteriostatic effects for partial-thickness burn wounds. It is useful as a dressing, as it is easier to apply and is less sticky than Manuka honey. However, for Gram-positive bacteria, Tualang honey is not as effective as usual care products, such as silver-based dressing or medical-grade honey dressing.	[90]
Tualang honey	In vitro	*Pediococcus acidilactici*, a lactic acid bacteria strain, was isolated from Tualang honey; it possessed antifungal activity against pathogenic candida species.	[93]
Gelam, Kelulut and Tualang	In vitro	Malaysian honey, namely Gelam, Kelulut, and Tualang, exhibited high antibacterial potency derived from total and non-peroxide activities, indicating that peroxide and other constituents are mutually important as contributing factors to the antibacterial property of honey.	[9]
Tualang honey	In vitro	Tualang honey demonstrated variable activities against different microorganisms but within the same range as with Manuka honey. Tualang showed higher antibacterial effect on *A. baumannii* and *S. maltophilia*.	[91]
Tualang honey	In vitro	Tualang honey showed antibacterial activity against *E. coli*, *S. typhi*, and *S. pyogenes*, with the most potent activity observed against *S. typhi*. However, it was merely ineffective against *S. sonnei*, *P. aeruginosa* and *S. aureus*.	[82]
Gelam and Tualang honey	In vitro	Gelam and Tualang honeys have been found to contain *Lactobacillus acidophilus*, with anti-microbial activity against multiple antibiotic-resistant *S. aureus*, *S. epidermis* and *Bacillus subtilis*. The anti-microbial compounds found were stable to heating, proteolytic enzyme treatment and pH adjustments.	[88]
Tualang honey	Human	Tualang honey administration, especially at 40 and 60 g daily doses for 6 months in asymptomatic patients with human immunodeficiency virus (HIV), reduced viral load and improved the CD4 counts and quality of life of these patients.	[92]

### 4.4. Anti-Inflammatory Properties

**Figure 4:**
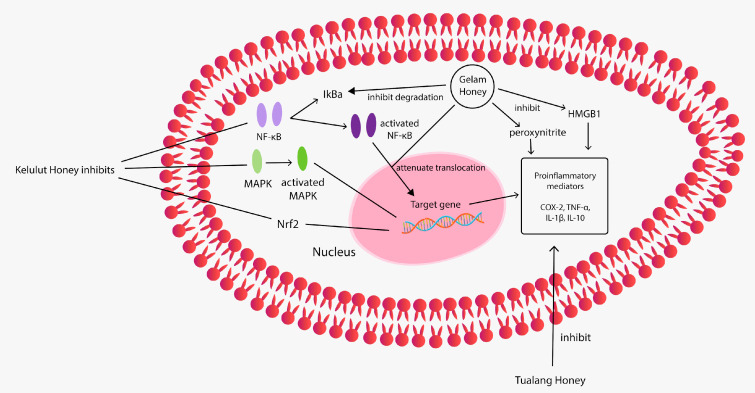
Anti-inflammatory mechanism of Tualang, Gelam and Kelulut honeys. TNF-α: tumor necrosis factor alpha; COX-2: cyclooxygenase-2; TNF- α: tumour necrosis factor alpha; IL-1β: interleukin-1 beta; IL-10: interleukin 10; NF-kB: nuclear factor kappa B; IκBα: NF-kappa-B inhibitor alpha; MAPK: mitogen-activated protein kinases; Nrf2: nuclear factor erythroid 2–related factor 2.

Tualang, Gelam, and Kelulut honeys demonstrated anti-inflammatory properties. Table 6 provides a summarisation of their anti-inflammatory properties and Figure 4 provides the pathway illustration. Gelam honey inhibited the production of proinflammatory mediators nitric oxide (NO), prostaglandin E2 (PGE2), TNF-α and IL-6 in carrageenan-induced acute paw oedema in rats. In this study, Gelam honey’s anti-inflammatory effect was similar to that of the anti-inflammatory drug indomethacin [95]. Another study showed that Gelam honey inhibited NO and PGE2 in inflammatory tissues of carrageenan-induced acute paw oedema in rats. The author claimed the phenolic compounds in Gelam honey to be responsible for the anti-inflammatory effects [96]. A study by Hussein et al. (2013) demonstrated Gelam honey’s inflammation inhibitory effects by attenuating NF-κB translocation to the nucleus and inhibiting IκBα degradation, which inhibits the production of proinflammatory mediators, including COX-2 and TNF-α [97]. Studies on the anti-inflammatory properties of Gelam honey were always consistent. For instance, the intravenous injection of Gelam honey inhibited lipopolysaccharide-induced endotoxemia in rats through the induction of heme oxygenase-1 and the inhibition of cytokines (TNF-α, IL-1β and IL-10), NO and high-mobility group protein B1 [98]. Besides, treatment with Gelam honey in lipopolysaccharide (LPS)-induced organ failure rat showed protective effects on organs by improving the organ blood parameters, reducing the infiltration of neutrophils and decreasing the myeloperoxidase activity and mortality compared with non-treatment. Thus, Gelam honey may have a therapeutic effect in protecting organs during inflammatory diseases [99]. Another mechanism for the anti-inflammatory properties of Gelam honey is through peroxynitrite synthesis inhibition during immune response in LPS-treated rats [100]. The anti-inflammatory properties of Gelam honey were further proven in two studies using asthma-induced mice and rabbits. Both studies reported that Gelam honey administration via oral gavage in mice and aerosolised Gelam honey in rabbits reduced airway inflammation by reducing the inflammatory cell [101,102]. Gelam honey-treated mice show similar improvement with the dexamethasone-treated group in terms of epithelium thickness, number of mast cells and mucus expression [101].

Tualang honey also demonstrated anti-inflammatory properties; pretreatment with Tualang honey reduced neuroinflammation by reducing the elevation of TNF-α, IL-1β, glial fibrillary acidic protein, allograft inflammatory factor 1 and COX-2 in the rat cerebral cortex, cerebellum and brainstem in kainic acid (KA)-induced status epilepticus rat [103]. Another study also reported a reduction in TNF-α, IL6 and IFN-γ in the brain homogenates of a Tualang honey-treated chronic stress rat model [104]. However, the anti-inflammatory properties of Tualang honey could not be translated to humans as a randomised controlled study demonstrated that Tualang honey supplementation has opposite effects on TNF-α and highly sensitive C-reactive protein, indicating the inconclusive effect of honey on inflammation amongst chronic smokers; thus, further studies are needed on other inflammatory markers or other study population [105]. Meanwhile, Kelulut honey has been reported to ameliorate the serum levels of CRP, TNF-α, IL-1β, IL-6, IL-8 and MCP-1 in LPS-induced chronic subclinical systemic inflammation in rats by modulating NF-κB, p38 MAPK and Nrf2 signaling [106].

**Table 6:** Summary of anti-inflammatory properties.

Type of Honey	Type of Study	Findings	References
Gelam honey	In vivo	Gelam honey showed anti-inflammatory effects by reducing rat paw oedema size and inhibiting the production of proinflammatory mediators nitric oxide (NO), prostaglandin E2 (PGE2), tumor necrosis factor alpha (TNF-α), and interleukin 6 (IL-6) in carrageenan-induced acute paw oedema in rats.	[95]
Gelam honey	In vivo	Gelam honey and its extracts inhibited oedema, pain, NO and PGE(2) in rats’ paws induced with carrageenan in the non-immune inflammatory and nociceptive model and lipopolysaccharide (LPS) in the immune-inflammatory model.	[96]
Gelam honey	In vivo	Gelam honey exhibited its inflammation inhibitory effects in carrageenan-induced rat paw inflammation by attenuating NF-kB translocation to the nucleus and inhibiting IkBa degradation, with a subsequent decrease in inflammatory mediators COX-2 and TNF-α.	[97]
Kelulut honey	In vivo	Kelulut honey protected against LPS-induced chronic subclinical systemic inflammation in rats mediated via amelioration of inflammation, oxidative stress and NF-κB, p38 MAPK and Nrf2 signalling.	[106]
Tualang honey	In vivo	Tualang honey treatment reduced TNF-α, IL6 and IFN-γ in brain homogenates of a chronic stress rat model.	[104]
Tualang honey	In vivo	Pre-treatment with Tualang honey reduced neuroinflammation by reducing the elevation of TNF-α, IL-1β, glial fibrillary acidic protein, allograft inflammatory factor 1 and COX-2 in the cerebral cortex, cerebellum and brainstem of kainic acid-induced status epilepticus rat.	[103]
Gelam honey	In vivo	Gelam honey alleviated the histopathological changes in a mouse model of allergic asthma.	[101]
Gelam honey	In vivo	Treatment with aerosolised Gelam honey reduced the number of airway inflammatory cells present in bronchoalveolar lavage fluid and inhibited goblet cell hyperplasia in a rabbit model of ovalbumin-induced chronic asthma.	[102]
Tualang honey	Human study	Tualang honey supplementation exhibited opposite effects on TNF-α and highly sensitive C-reactive protein amongst chronic smokers.	[105]
Gelam honey	In vivo	Gelam honey inhibited lipopolysaccharide-induced endotoxemia in rats through the induction of heme oxygenase-1 and the inhibition of cytokines, nitric oxide and high-mobility group protein B1.	[98]
Gelam honey	In vivo	LPS-induced organ failure rats treated with Gelam honey demonstrated protection on organs through improved organ blood parameters, reduced infiltration of neutrophils, decreased myeloperoxidase activity and reduced mortality compared with untreated rabbits.	[99]
Gelam honey	In vivo	Gelam honey showed anti-inflammatory properties through peroxynitrite synthesis inhibition during immune response in LPS-treated rats.	[100]

### 4.5. Anti-Diabetic Properties

Only Tualang and Kelulut honeys were found to have an anti-diabetic effect in three animal studies [50,107,108] and two human studies [109,110], as summarised in Table 7. Studies using a diabetic model of rat showed that Tualang honey possessed moderate hypoglycaemic effect [50], improved liver enzyme profile [107], and possessed a synergetic benefit on glycaemic and metabolic profiles when administered together with metformin or glibenclamide [108].

Tualang honey was found to have intermediary glycemic index values of 65 ± 7 [110]. Hussain et al. (2012) reported that supplementation of 20 g/day Tualang honey for 4 months in healthy postmenopausal women caused a significant increase in fasting blood sugar (FBS) [111]. However, by prolonging the treatment to 12 months, a decrease in FBS levels was reported in healthy and diabetic postmenopausal women [112]. Wahab et al. (2018) suggested a differential effect of honey on glucose metabolism for short- and long-term use. [112]. Recent findings by Rashid et al. (2019) supported a prior claim that the ingestion of Kelulut honey daily for 30 days has no significant effect on fasting glucose and fasting lipid profile in patients with impaired fasting blood glucose [109]. The authors suggested short-duration ingestion of Kelulut honey as the factor for the insignificant result. By contrast, one in vivo study showed that supplementation of Kelulut honey for the last 35 days in a 16-week high-carbohydrate high-fructose diet fed in rats decreased the fasting blood glucose compared with non-supplemented control [113]. However, the effect of Tualang honey as an anti-diabetic is unclear. Future studies should extend knowledge on the anti-diabetic properties of Tualang honey on patients with diabetes to reinforce the anti-diabetic ability of Tualang honey, as the animal studies discussed above [50,107,108] have successfully proven that Tualang honey possesses anti-diabetic effect in animals.

Although the anti-diabetic effect of honey may sound paradoxical as it contains a high content of sugar, several studies proved it otherwise. The honey from Nigeria has been reported to have an anti-hyperglycaemic effect in diabetic rats [114]. Moreover, the honey from Iran was reported to cause a mild reduction in FBS by 4.2% among overweight adults [115]. The fructose, oligosaccharides, antioxidants and trace minerals present in honey may contribute to its glucose-lowering effect [114]. Disaccharide trehalulose has been recently revealed as the main component of Kelulut honey with a low insulinemic and glycaemic index [1]. However, contradictory results of anti-diabetic properties of honey were also reported, as the honey from Iran increased the HbA1c level after 8 weeks of honey supplementation amongst patients with type 2 diabetes [116]. Thus, studies to discover the relation between honey and glucose metabolism in humans are needed to enlighten the natural characteristic of honey, whether it is beneficial or contraindicated in patients with diabetes.

**Table 7:** Summary of anti-diabetic properties.

Type of Honey	Type of Study	Findings	References
Tualang honey	In vivo	Tualang honey produced a moderate hypoglycaemic effect and ameliorated oxidative stress in the kidneys of streptozotocin-induced diabetic rats.	[50]
Tualang honey	In vivo	Tualang honey demonstrated hepatoprotective effect in diabetic rats by reducing aspartate aminotransferase (AST), alanine aminotransferase (ALT) and alkaline phosphatase (ALP) activities.	[107]
Tualang honey	In vivo	Combination of glibenclamide or metformin with Tualang honey improved glycaemic control and provided additional metabolic benefits not achieved with either glibenclamide or metformin alone.	[108]
Kelulut honey	Human study	Thirty g of Kelulut honey daily intake for 30 days caused no changes in fasting glucose, fasting lipid profiles, body mass index (BMI), waist circumference, blood pressure, total cholesterol, triglyceride, high-density lipoprotein (HDL) and low-density lipoprotein (LDL) in patients with impaired fasting glucose.	[109]
Tualang honey	Human study	Tualang honey is an intermediate glycaemic index food (GI = 65 ± 7).	[110]

### 4.6. Anti-Obesity Properties

Only Gelam and Kelulut honeys have been found to show anti-obesity properties in animal studies [117,118,119], as provided in Table 8. Supplementation of Kelulut honey yielded a higher reduction in body mass index (BMI), the percentage of body weight gain, adiposity index, and relative organ weight in a high-fat diet-induced obese rat model than that of orlistat, an anti-obesity drug [117]. Meanwhile, another study has shown that Kelulut honey could prevent changes, such as high omental fat mass, serum triglyceride, systolic blood pressure, diastolic blood pressures, adipocyte area and adipocyte perimeter, caused by metabolic syndrome-induced rats [119].

Gelam honey has been shown to possess lipid-lowering and anti-oxidative capabilities in obesity-induced rats and weight-reducing ability compared with Acacia honey. However, honey showed better effects than orlistat, as the orlistat group showed hepatotoxicity effects [118].

A study conducted by Iranian researchers found that honey supplementation in humans also reduced body weight, body fat and total cholesterol [115]. As of now, Tualang honey has not been demonstrated to have an anti-obesity effect. Meanwhile, Gelam and Kelulut honeys have not been explored for their anti-obesity potential in humans.

**Table 8:** Summary of anti-obesity properties.

Type of Honey	Type of Study	Findings	References
Kelulut honey	In vivo	Kelulut honey treatment showed improved BMI, percentage of body weight gain, adiposity index, relative organ weight, LDL, HDL and hepatoprotective effect in high-fat diet-induced obese rats.	[117]
Gelam honey	In vivo	Reductions in excess weight gain, adiposity index levels, plasma glucose, triglycerides, cholesterol, plasma leptin and resistin, liver enzymes, renal function test and relative organ weight were found in Gelam honey-treated groups compared with rats fed with the high-fat diet.	[118]
Kelulut honey	In vivo	Kelulut honey supplementation significantly prevented changes in omental fat mass, serum triglyceride, systolic blood pressure, diastolic blood pressures, adipocyte area and adipocyte perimeter induced by high-carbohydrate and high-fat diet in rat.	[119]

### 4.7. Wound-Healing Properties

**Figure 5:**
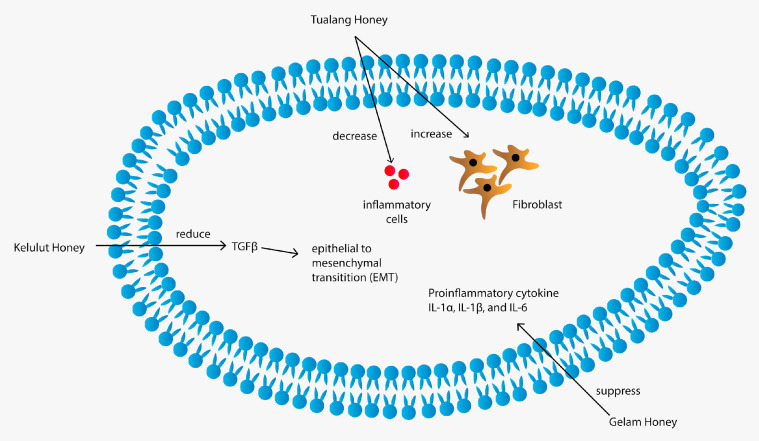
Wound-healing mechanism of Tualang, Gelam and Kelulut honeys. TGFβ: transforming growth factor beta; IL-1α: interleukin-1α; IL-1β: interleukin-1β; IL-6: interleukin-6.

Honey has been used to treat wounds beginning from ancient times [120]. This practice was rooted primarily in tradition and folklore when investigators began to explore its medicinal potential [121]. The honey from different geographical areas has been reported to have considerable therapeutic effects on chronic wounds, ulcers and burns [122]. A study reported that honey has almost equal or slightly superior effects compared with conventional treatments for acute wounds and superficial partial-thickness burns [3]. Tualang, Gelam and Kelulut honeys have been demonstrated to have wound-healing properties, as listed in Table 9. Their wound-healing mechanism is provided in Figure 5.

In two studies, Tualang honey was compared with hydrofibre, hydrofibre silver or chitosan gel on full-thickness burn wounds in rats inoculated with *Pseudomonas aeruginosa*, *K. pneumoniae* or *Acinetobacter baumannii*. The first study by Khoo et al. (2010) found that the Tualang honey-treated group had more improved wound contraction and control over *P. aeruginosa* growth than the hydrofibre- and hydrofibre silver-treated groups [123]. A study by Sukur et al. (2011) showed that topical application of Tualang honey on burn wounds contaminated with *P. aeruginosa* and *A. baumannii* provided a faster healing rate than chitosan gel or hydrofibre silver treatment [124]. Meanwhile, another study showed that the oral treatment of Tualang honey enhanced anastomotic wound healing in large bowel anastomosis in rats by increasing the number of fibroblasts and decreasing inflammatory cells, leading to increased wound strength [125]. A clinical trial looking into Tualang honey’s effect on promoting the post-tonsillectomy healing process has been conducted by comparing sultamicillin-treated group and sultamicillin + Tualang honey-treated group. Tualang honey was topically administered intraoperatively and 4 ml orally three times daily for 7 days after surgery. The results obtained showed that the healing process was much faster in the Tualang honey + sultamicillin-treated group [126]. The multiple studies discussed above found Tualang honey to yield better wound-healing improvement than the current antibiotic or topical treatment, which could suggest the use of Tualang honey as a wound dressing.

Gelam honey has also been demonstrated to have wound-healing properties. A study reported that Gelam honey-dressed wounds healed faster with less scab and only thin scar formations than the untreated, saline-treated and intrasite gel-treated full-thickness excisional wounds in rats [127]. Zohdi et al. (2012) developed a Gelam honey-based hydrogel. They found that the application of Gelam honey hydrogel dressings significantly increased wound closure and accelerated the rate of re-epithelialisation compared with control hydrogel and OpSite film dressing in deep partial-thickness burn wounds in rats [128]. The application of Gelam honey-based hydrogel decreased the inflammatory response as seen by the suppressed expression of proinflammatory cytokines IL-1α, IL-1β and IL-6 [128]. In another study, Gelam honey was shown to promote in vitro corneal fibroblast wound healing, where corneal fibroblast cultured with Gelam honey demonstrated faster wound closure than the control group [129]. Moreover, two studies have shown that Gelam honey at a concentration of 0.0015% promoted ex-vivo corneal keratocytes and corneal epithelial cell proliferation whilst preserving their phenotypical features [130,131]. These findings could suggest Gelam honey formulation gel for topical treatment in wound healing for humans.

Wound healing is associated with keloid scar formation. Keloid scar formation is due to the excessive induction of EMT by TGFβ in keratinocytes. In a study, treatment of 0.0015% Kelulut honey has been demonstrated to reduce the TGFβ-induced EMT in human primary keratinocytes, indicating Kelulut honey’s therapeutic potential in preventing keloid scar formation [132]. Rats pretreated with Kelulut honey showed significantly reduced total area of ulcer and ulcer index compared with an untreated ethanol-induced gastric ulcer rat [133]. Honey is generally reported to have remarkable effects compared with conventional treatments for acute wounds, superficial partial-thickness burns and infected post-operative wounds [3,4].

**Table 9:** Summary of wound-healing properties.

Type of Honey	Type of Study	Findings	References
Gelam honey	In vivo	Application of Gelam honey hydrogel dressings enhanced wound closure and accelerated the rate of re-epithelialisation, with a significant decrease in inflammatory response and expression of proinflammatory cytokines (IL-1α, IL-1β and IL-6).	[128]
Tualang honey	In vivo	Tualang honey demonstrated enhanced control of *Pseudomonas aeruginosa* and wound contraction effects on full-thickness burn wound in vivo.	[123]
Gelam honey	In vivo	Gelam honey dressing on excisional wounds accelerated the process of wound healing, with less scab and only thin scar formations.	[127]
Gelam honey	Ex vivo	Gelam honey (0.0015%) promoted ex-vivo corneal keratocyte proliferation whilst preserving phenotypical features.	[130]
Gelam honey	Ex vivo	Gelam honey promoted the proliferative phase of corneal reepithelialisation whilst preserving phenotypical features.	[131]
Gelam honey	In vitro	Gelam honey promoted ex-vivo corneal fibroblast wound healing.	[129]
Tualang honey	in vivo	Topical application of Tualang honey on full-thickness burn wounds contaminated with *P. aeruginosa* and *A. baumannii* provided the fastest rate of healing amongst Chitosan gel or hydrofibre silver treatments.	[124]
Tualang honey	In vivo	Oral treatment with Tualang honey enhanced anastomotic wound healing by increasing the number of fibroblasts and decreasing inflammatory cells, leading to increased wound strength.	[125]
Kelulut honey	In vivo	Rats pre-treated with Kelulut honey showed significantly reduced total area of ulcer and ulcer index compared with untreated ethanol-induced gastric ulcer rats.	[133]
Kelulut honey	In vitro	Kelulut honey (0.0015%) reduced TGFβ-induced EMT in human primary keratinocytes, indicating its therapeutic potential in preventing keloid scar formation.	[132]
Tualang honey	Human	Tualang honey enhanced the healing process in patients who underwent tonsillectomy.	[126]

### 4.8. Effects on Cardiovascular System

Only Tualang honey was reported to affect the cardiovascular system in three animal studies [134,135,136], one study of combined in-vivo and in vitro techniques [137] and two human studies [111,112]. The studies are listed in Table 10. All animal studies reported a positive effect of Tualang honey on the cardiovascular system, whilst human studies reported varied results. Khalil et al. (2015) showed that the pretreatment of ischaemic rats with Tualang honey yielded significant protective effects on cardiac troponin I, triglycerides and total cholesterol. The authors contributed to improving the endogenous antioxidant enzyme activity and inhibition of lipid peroxidation by Tualang honey [134]. Two studies reported that Tualang honey supplementation considerably reduced elevated systolic blood pressure via amelioration of spontaneous hypertensive rats [135,136]. Devasvaran et al. (2019) stated that in vitro Tualang honey could inhibit H_2_O_2_-induced vascular hyperpermeability in vitro and in vivo by suppressing adherence junction protein redistribution via calcium and cAMP [137].

A randomised controlled trial comparing the effects of Tualang honey and hormone replacement therapy for 4 months showed no demonstrable effects on blood pressure measurement, BMI and waist circumference. No significant difference was found in the lipid profile, blood sugar profile and bone density between the two groups. However, a statistically significant increase in total cholesterol, low-density lipoprotein cholesterol (LDL-C) and FBS levels was observed in the honey-treated group at 4 months of the study compared with the baseline value [111]. A study on humans compared the effect of 20 g Tualang honey only and honey cocktail (a mixture of Tualang honey, beebread and royal jelly) for 12 months. Supplementation of Tualang honey only showed a significant effect in lowering diastolic blood pressure and FBS compared with supplementation of honey cocktail. However, no demonstrable effect of Tualang honey on the lipid profiles and anthropometric measurements was found [112].

**Table 10:** Summary of the effects on cardiovascular system.

Type of Honey	Type of Study	Findings	References
Tualang honey	In vitro and in vivo	Tualang honey could inhibit H_2_O_2_-induced vascular hyperpermeability in vitro and in vivo by suppressing adherence junction protein redistribution via calcium and cyclic adenosine monophosphate (cAMP).	[137]
Tualang honey	In vivo	Pre-treatment with Tualang honey demonstrated cardioprotective effects on isoproterenol-induced myocardial infarction in rats by improving cardiac marker enzyme and ameliorating oxidative stress.	[134]
Tualang honey	In vivo	Tualang honey supplementation considerably reduced elevated systolic blood pressure via amelioration of oxidative stress in spontaneously hypertensive rats’ kidneys.	[135]
Tualang honey	In vivo	Tualang honey ameliorated oxidative stress and reduced elevated blood pressure in spontaneous hypertensive rats but not in diabetic rats.	[136]
Tualang honey	Human study	Tualang Honey supplementation showed a superior effect in lowering diastolic blood pressure and fasting blood sugar over honey cocktail (a mixture of honey, beebread and royal jelly) in postmenopausal women.	[112]
Tualang honey	Human study	Four months of Tualang honey supplementation in postmenopausal women showed no changes in blood pressure, waist circumference, BMI, lipid profile, blood sugar and bone density compared with low-dose hormone replacement therapy.	[111]

### 4.9. Effects on Reproductive System

Tualang, Gelam and Kelulut honeys demonstrated benefits toward male and female reproductive systems. All of these benefits are listed in Table 11. Findings from animal studies have shown that Tualang honey is beneficial for the postmenopausal syndrome. Amongst the benefits are preventing uterine atrophy, increasing bone density and suppressing body weight elevation [138]. Ovariectomised rats that received Tualang honey showed more trabecular bone structure improvements than those who received calcium supplements [139]. These findings may suggest the potential of Tualang honey as an alternative to hormone replacement therapy for postmenopausal women.

Moreover, Tualang honey appeared to have a protective role against bisphenol-A (BPA)-induced toxicity in rat uterus by improving the morphological abnormalities, reducing lipid peroxidation and normalising ERα, ERβ and C3 expression distribution in the rat uterus [140]. The same protective effect was also exhibited in BPA-induced ovarian toxicity, where Tualang honey reduced the morphological abnormalities of the ovarian follicles and improved the normal estrous cycle [141].

In the male reproductive system, Tualang honey at a dose of 1.2 g/kg/day is the most effective dose to alter the male reproductive parameter as it significantly increased epididymal sperm count [142]. This finding is parallel with the result of the other two studies using 1.2 g/kg/day of Tualang honey treatment that successfully demonstrated Tualang honey’s benefit on the male reproductive system [143,144]. In response to impaired sexual behavior and fertility, Tualang honey was revealed to have a protective effect against cigarette smoke-induced impaired sexual behavior and fertility in male rats by significantly increasing intromission and ejaculation and increasing mating fertility indices [143]. In a separate study, Tualang honey supplementation during prenatal restraint stress significantly increased testis and epididymis weights. It improved the percentages of abnormal spermatozoa and sperm motility in male rat offspring [144]. In humans, a randomised control study showed that the effect of Tualang honey amongst oligospermic males was comparable with that of Tribestan in improving sperm concentration, motility and morphology [145]. Apart from Tualang honey, Kelulut honey has been shown to benefit the male reproductive system. In streptozotocin-induced diabetic rats, Kelulut honey treatment prevented sperm and testicular oxidative damage by improving sperm quality and increasing the spermatozoa and spermatogenic cells [55]. One possible mechanism is the anti-oxidative properties that are mostly attributed to the phenolic and flavonoid contents of honey. For support, Budin et al. (2017) reported increased SOD and GSH activities and decreased PC and MDA levels in sperm and testis of streptozotocin-induced diabetic rats [55].

Tualang honey has been shown to have various advantages for female and male reproductive systems. However, future works should explore the chemical constituents responsible for the reproductive effect of Tualang and Kelulut honeys. Meanwhile, Gelam honey has not been explored for its effect on the reproductive system.

**Table 11:** Summary of the effects on reproductive system.

Type of Honey	Type of Study	Findings	References
Tualang honey	In vivo	The Tualang honey-treated group showed more trabecular bone structure improvements than experimental postmenopausal rats who received calcium.	[139]
Tualang honey	In vivo	Tualang honey prevented uterine atrophy, increased bone density and suppressed increased body weight in ovariectomised rats.	[138]
Kelulut honey	In vivo	Kelulut honey prevented damage of sperm and testis in diabetic rats.	[55]
Tualang honey	In vivo	Tualang honey protected the uterus from bisphenol-A (BPA)-induced toxicity.	[140]
Tualang honey	In vivo	Tualang honey demonstrated a protective effect against cigarette smoke-induced impaired sexual behaviour and fertility in male rats.	[143]
Tualang honey	In vivo	Tualang honey at a dose of 1.2 g kg daily increased epididymal sperm count without affecting spermatid count and reproductive hormones.	[142]
Tualang honey	In vivo	Tualang honey supplementation during pregnancy reduced the adverse effects of prenatal restraint stress on reproductive organ weight and sperm parameters in male rat offspring.	[144]
Tualang honey	In vivo	Tualang honey reduced BPA-induced ovarian toxicity by reducing the ovarian follicles’ morphological abnormalities and improving the normal oestrous cycle.	[141]
Tualang honey	Human study	The Tualang honey effect amongst oligospermic males was comparable with that of Tribestan in improving sperm concentration, motility and morphology.	[145]

### 4.10. Effects on Nervous System

The effect of Tualang, Gelam and Kelulut honeys on the nervous system is summarised in Table 12. Tualang honey has been reported in several studies to improve memory and reduce depressive-like behavior in animals and humans. In noise stress-induced memory deficits in aged rats, Tualang honey administration was shown to protect against memory decline via enhancement of medial prefrontal cortex and hippocampal morphology, possibly through the reduction of brain oxidative stress and the up-regulation of brain-derived neurotrophic factor (BDNF) concentration and cholinergic system [146]. These findings were further corroborated by Azman et al. (2015), who showed that Tualang honey administration improved memory performance and decreased depressive-like behavior in rats exposed to loud noise stress [147].

Several clinical studies have found that oestrogen treatment has a protective effect on aging women’s cognitive decline [148]. In a study on stressed ovariectomised rats, Tualang honey or 17β-estradiol treatment has been shown to demonstrate antidepressive-like effects, possibly via restoration of the hypothalamic-pituitary-adrenal axis and enhancement of the BDNF concentration [149]. In the same set of studies, rats administered with Tualang honey or 17β estradiol showed improved short-term and long-term memory and enhanced neuronal proliferation of hippocampal CA2, CA3 and dentate gyrus (DG) regions compared with untreated stressed ovariectomised rats [150]. In addition, Al-Rahbi et al. (2014) demonstrated the anti-anxiety effect of Tualang honey in ovariectomised rats with the improvement of its oxidative stress status [151]. A randomised controlled trial comparing Tualang honey supplementation and oestrogen plus progestin therapy demonstrated that postmenopausal women who received Tualang honey showed improvements in their immediate memory but not in immediate memory after the interference and delayed recall as oestrogen plus progestin therapy. However, this finding is comparable with the improvement seen in women receiving oestrogen plus progestin treatment [152]. These animal and human studies may serve as a basis to propose Tualang honey as a potential supplement with comparable effects as oestrogen and with no side effects in postmenopausal women.

Tualang honey has been reported in several studies to exert a neuroprotective effect through its antioxidant properties. In a study of kainic acid KA-treated rats, pretreatment with Tualang honey reduced the KA-induced neuronal degeneration in the piriform cortex but failed to prevent the occurrence of KA-induced seizures. In addition, KA-induced rats showed increased locomotor activity and hyperactivity, which were attenuated by Tualang honey pretreatment [103]. A recent study reported that Tualang honey pretreatment significantly attenuated an increase in lipid peroxidation level and decreased the total antioxidant status level induced by KA treatment in the rat cerebral cortex. These findings indicated that pretreatment with Tualang honey has therapeutic potential against KA-induced oxidative stress and neurodegeneration through its antioxidant effect [153]. In a separate study, Tualang honey improved the oxidative stress status, spinal cord morphology and nociceptive behavior in offspring of prenatally stressed rats [154]. The anti-nociceptive effect of Tualang honey was again demonstrated in another study that showed a similar reduction in pain behavior as the prednisolone-treated group [155]. Finally, Tualang honey pretreatment was shown to be comparable with ubiquinol in protecting the rat midbrain, lung toxicity and oxidative stress against repeated paraquat exposure [156].

Regarding Kelulut honey, a study on metabolic disease-induced rats showed that the Kelulut honey-treated group exhibited less anxious behavior and demonstrated significant memory retention than the untreated metabolic disease-induced group [113]. However, the underlying mechanism of Kelulut honey in showing this benefit has yet to be discovered by researchers.

**Table 12:** Summary of the effect on nervous system.

Type of Honey	Type of Study	Findings	References
Kelulut honey	In vivo	Kelulut honey reduced serum triglyceride and LDL and normalised blood glucose levels in metabolic disease-induced rats. Behavioural studies showed lessened anxious behaviour and enhanced memory retention.	[113]
Tualang honey	In vivo	Tualang honey improved memory performance and decreased depressive-like behaviour in rats exposed to loud noise stress.	[147]
Tualang honey	In vivo	Tualang honey or 17β-oestradiol treatment demonstrated anti-depressive-like effects, possibly via restoration of the hypothalamic-pituitary-adrenal axis and enhancement of the brain-derived neurotrophic factor (BDNF) concentration in stressed ovariectomised rats.	[149]
Tualang honey	In vivo	Administration of either oestrogen or Tualang honey significantly decreased anxiety-like behaviour in stressed ovariectomised rats, with improved oxidative stress status.	[151]
Tualang honey	In vivo	Tualang honey and oestrogen treatments improved short-term and long-term memory and enhanced the neuronal proliferation of hippocampal CA2, CA3 and DG regions in stressed ovariectomised rats.	[150]
Tualang honey	In vivo	Pre-treatment with Tualang honey reduced kainic acid-induced neuronal degeneration, hyperactivity and oxidative stress but failed to prevent seizures.	[153]
Tualang honey	In vivo	Tualang honey protected against memory decline due to noise stress exposure and ageing via enhancement of medial prefrontal cortex and hippocampal morphology, possibly secondary to a reduction in brain oxidative stress and upregulation BDNF concentration and the cholinergic system.	[146]
Tualang honey	In vivo	Treatment with Tualang honey ameliorated the toxic effects induced by repeated paraquat exposure observed in midbrain and lungs. Tualang honey effect was comparable to that of ubiquinol.	[156]
Tualang honey	In vivo	Tualang honey improved oxidative stress status and spinal cord morphology and nociceptive behaviour in the offspring of prenatally stressed rats.	[154]
Tualang honey	In vivo	Pre-treatment with Tualang honey (1.2 and 2.4 g/kg) and prednisolone (10 mg/kg) reduced pain responses in the rat.	[155]
Tualang honey	In vivo	Postmenopausal women who received Tualang honey showed improvement in their immediate memory but not in immediate memory after interference and delayed recall. This finding was comparable to the improvement observed in women receiving oestrogen plus progestin therapy.	[152]

## 5. Conclusions

Tualang, Gelam and Kelulut honeys were extensively demonstrated to have various health benefits to multiple diseases and systems. Clinical trials, especially in wound healing, showed the superior benefit of Tualang honey as a wound dressing over conventional dressing. However, clinical trials using Tualang, Gelam, and Kelulut honeys are still needed to add to the results because convincing effects are still yet to be finalised. In addition, the mechanism of action of these honeys was not clear, especially in human studies, thereby leaving an area to be discovered. In comparison, Tualang and Gelam honeys have been well researched compared with Kelulut honey. Thus, future studies may focus more on the potential benefit of Kelulut honey. This study also revealed that Tualang, Gelam and Kelulut honey has excellent preclinical potential in multiple diseases and physiological systems that could steer future research to thoroughly explore honey as an efficient and proven superfood to be optimised for the benefit of humanity.

## Data Availability

Data sharing is not applicable to this article.
